# Impaired Cerebral Haemodynamics in Vascular Depression: Insights From Transcranial Doppler Ultrasonography

**DOI:** 10.3389/fpsyt.2018.00316

**Published:** 2018-07-16

**Authors:** Valentina Puglisi, Alessia Bramanti, Giuseppe Lanza, Mariagiovanna Cantone, Luisa Vinciguerra, Manuela Pennisi, Lilla Bonanno, Giovanni Pennisi, Rita Bella

**Affiliations:** ^1^IRCCS Centro Neurolesi “Bonino Pulejo”, Messina, Italy; ^2^Istituto di Scienze Applicate e Sistemi Intelligenti, Messina, Italy; ^3^Oasi Research Institute, IRCCS, Troina, Italy; ^4^Spinal Unit, Ospedale Cannizzaro, Catania, Italy; ^5^Department of Surgery and Medical-Surgical Specialties, University of Catania, Catania, Italy; ^6^Section of Neurosciences, Department of Medical and Surgical Sciences and Advanced Technology, University of Catania, Catania, Italy

**Keywords:** transcranial doppler, neurosonology, geriatric depression, cerebrovascular disease, hypoperfusion, leukoaraiosis

## Abstract

**Introduction:** Late-life depression is a well-known risk factor for future dementia. Increasing evidences also show a link between cerebral hypoperfusion and neurodegeneration, although data on Transcranial Doppler ultrasonography (TCD)-derived measures in patients with “Vascular Depression” (VD) are lacking. The aim of this study was to assess and correlate TCD parameters with cognitive function and severity of subcortical ischemic vascular disease in a sample of VD patients.

**Methods:** Seventy six patients (mean age 72.5 ± 5.3 years; 53.9% females) met the DSM-5 diagnostic criteria for unipolar major depression. Mean blood flow velocity (MBFv) and pulsatility index (PI) were recorded from the middle cerebral artery. Quantification of depressive symptoms (17-item Hamilton Depression Rating Scale –HDRS), neuropsychological test evaluating frontal lobe abilities (Stroop Color-Word test interference—Stroop T), and white matter lesions (WMLs) load according to the Fazekas visual score were also assessed.

**Results:** WMLs severity was mild in 20 patients (group I), moderate in 32 (group II), and severe in 24 (group III). The groups were comparable in terms of clinical features, vascular risk factors profile, and HDRS score, whereas Stroop T score was worse in group III. An increased PI and a reduced MBFv were found in VD patients with severe WMLs. According to the regression analysis, a reduced MBFv was independently and significantly associated with depressive symptoms and executive dysfunction, even after adjusting for demographic features and vascular risk factors. Similarly, an independent and significant association was observed between the increase of PI and both Stroop T and WMLs severity.

**Conclusions:** A TCD profile of low perfusion and high vascular resistance in VD patients suggests a diffuse cerebrovascular pathology likely arising from the small vessels and then extending to larger arteries. Hemodynamic dysfunction might play a pathogenic role in the development of cognitive impairment in patients with late-life depression and subcortical ischemic vascular disease. TCD represents a valuable tool in the early detection, assessment, and management of VD patients at risk for dementia.

## Introduction

The “Vascular depression (VD) hypothesis” typically identifies patients with a “depression-executive dysfunction syndrome of the late life” (1), and is clinically characterized by psychomotor retardation, limited depressive ideation, and lack of interest and insight. Moreover, several neuroimaging studies in late-life depression (LLD) widely report an increased prevalence and severity of white matter lesions (WMLs) in frontal subcortical areas modulating mood, affect, and cognition (2, 3). Growing evidences also demonstrated that LLD may contribute to the development of dementia, being it considered both as a risk factor and a prodromal symptom of cognitive disorders (4–6). This increased risk is associated with depressive symptoms severity, presence of multiple vascular co-morbidities, and structural brain changes (namely, ischemic WMLs) (7, 8).

A number of studies showed that impairment of brain perfusion and cerebral blood flow (CBF) can precede the clinical onset of dementia (9, 10), thus leading to the hypothesis that cerebral hypoperfusion is one of the mechanisms by which vascular disease may contribute to neurodegeneration (11, 12). Moreover, cerebral hypoperfusion was demonstrated to be either a risk or an aggravating factor of dementia, being not only an epiphenomenon of brain tissue loss but actively promoting, initiating, or accelerating neurodegenerative disorders (13, 14).

Early changes in the intracranial blood vessel wall can be reliably identified by ultrasound techniques, which allow to detect even minimal or subclinical changes (15). As recently reviewed (16), ultrasound can evaluate structural and functional changes of the cerebral vessels that contribute to hypoperfusion in dementing disorders. In particular, Transcranial Doppler ultrasonography (TCD) is a non-invasive, inexpensive, and portable imaging modality with a good inter-observer reliability (17). TCD, through the evaluation of the mean blood flow velocity (MBFv) and the Gosling's Pulsatility Index (PI), is able to assess the cerebral hemodynamics of the main cerebral arteries. While MBFv is a relative measure of the arterial perfusion integrity, PI reflects the resistance of the small vessels and the intracranial compliance (18, 19).

Up to now, TCD studies have largely focused on the evaluation of hemodynamics in patients with Mild Cognitive Impairment, Alzheimer's disease, and vascular dementia (VaD) (20–22); conversely, no study is currently available in VD. The aim of the present investigation was to assess the association between changes of TCD parameters and cognitive decline in non-demented elderly patients with LLD and neuroimaging evidence of subcortical ischemic vascular disease (SIVD). We hypothesized that TCD-related measures of hypoperfusion might be considered as a predictive factor of cognitive decline.

## Materials and methods

### Participants

This was a cross-sectional study that has consecutively recruited all elderly patients (65 years or later) with depressive symptoms and neuroimaging evidence of SIVD attending the Cerebrovascular Disease Center of the University of Catania (Italy), from November 2016 to September 2017.

All participants performed a brain Magnetic Resonance Imaging (MRI) and fulfilled the MRI criteria (23) for SIVD with predominant WMLs. These represent a modification of the NINDS-AIREN criteria as a new research criteria for subcortical VaD. As such, they overcome the limitations of the clinical criteria for the diagnosis of VaD and are widely used in clinical trials (23). The participants were required to have a score >7 on the 17-item Hamilton Depression Rating Scale (HDRS) (24) and to meet the DSM-5 diagnostic criteria for unipolar major depression. Exclusion criteria were: other neurological disorders (i.e., Parkinson's disease, stroke, transient ischemic attack, dementia, head trauma, epilepsy, etc.); family or personal history of major depression with a young age of onset and other psychiatric diseases; acute medical illness or endocrinopathies associated with depression; alcohol or drug abuse; Mini Mental State Examination (MMSE) score ≤ 24 (25); conditions precluding MRI execution; ultrasound evidence of carotid or vertebral extracranial artery stenosis ≥50%; ultrasound evidence of intracranial artery stenosis; absence of an adequate transtemporal windows for TCD examination, bilaterally. The absence of depressed mood episodes prior to the recruitment was carefully assessed through a multidimensional interview of both patients and their relatives and General Practitioners. Moreover, the Structured Clinical Interview for DSM-5 (SCID-5) was used to support the presence of the current episode and to exclude any previous episode (26). Finally, an accurate check of patients' medical documents (including past clinical records) has been carried out before the enrolment.

This study was carried out in accordance with the recommendations of the Declaration of Helsinki of 1964 and its later amendments. The protocol was approved by the Ethics Committee of the “Azienda Ospedaliero-Universitaria Policlinico Vittorio Emanuele” of Catania, Italy. All subjects gave written informed consent prior to the entry in the study.

### Assessment

Clinical and demographic assessment included: age, gender, education, presence of cardio- and cerebrovascular risk factors, personal or family history of depression, presence of neurological signs and symptoms. The SCID-5 was used for diagnostic evaluation, whereas the severity of depressive symptoms was rated by means of HDRS, which has proved to be a valid tool for the quantitative assessment of mood symptoms among participants with VD and VaD (27–29). The neuropsychological tests included a screening tool of cognitive impairment (MMSE) and the assessment of executive functions by means of the Stroop Color-Word Test interference (normative values collected from an Italian population sample; Stroop T score ≤ 36.92 s) (30). The battery was performed by a trained neurologist (GL) expert in psychometric evaluation of the elderly with vascular cognitive impairment and neuropsychiatric disorders.

All patients were treated for their vascular risk factors with anti-platelet or anticoagulant medications (aspirin, clopidogrel, warfarin), anti-hypertensive drugs (angiotensin-converting enzyme inhibitors, angiotensin II receptor antagonist, diuretics, calcium channel blockers if necessary), cholesterol lowering medications (statins), and oral antidiabetic drugs or insulin. Depression was treated with Selective Serotonin Reuptake Inhibitors, or Serotonin and Noradrenaline Reuptake Inhibitors, or Serotonin Modulator and Stimulator (vortioxetine). In addition, patients were not withdrawn from their antidepressants, the doses of which remained stable before and during the study. Among nine patients with atrial fibrillation, six suffered from a paroxysmal form of the arrhythmia, which was not present at the time of TCD examination; conversely, two patient in the group III and one in the group I had permanent atrial fibrillation with low frequency rate.

### Brain MRI and WMLs quantification

Brain MRI was acquired with a 1.5 T General Electric system. The protocol included the T1-, T2-, proton density-weighted, and the Fluid Attenuated Inversion Recovery scans; slice thickness was 5 mm with 0.5 mm slice gap. The severity of deep WMLs was graded according to the visual scale of Fazekas: 0 = absence; 1 = punctuate foci; 2 = partially confluent foci; 3 = large confluent areas (31). Accordingly, patients were divided into three group: group I (Fazekas score = 1), group II (Fazekas score = 2), and group III (Fazekas score = 3), in order to obtain a more powerful association between TCD parameters and the different severity of SIVD.

### TCD protocol

Given that the goal of this study was the examination of cerebral hemodynamics, we have used TCD that, as known, specifically allows to record blood flow velocities from the basal cerebral arteries through the skull, based on the depth of the sample volume, the position of the transducer, and the flow direction (32). TCD (Compumedics DWL, Multi-Dop® X digital, Singen, Germany) was performed by the same experienced operator (RB), who was blind to the patient's neuropsychological performance and WMLs load. The TCD setting was as follows: intensity spatial-peak temporal-average: 420 mW/cm^2^; sweep step: 5 [1 s/div]; sample volume: 12 mm in length; thermal index: 1.9. After a 30-s stabilized recording period, TCD measurements, lasting at least for 10 cardiac cycles, were acquired (33).

Blood flow velocity of the proximal tract (M1) of the middle cerebral artery (MCA) was recorded with a handheld 2 MHz DWL® Ultrasound Probes PW, through the temporal bone window, under resting conditions and at the depth that provided the best signal (50–60 mm). Peak systolic velocity, end-diastolic velocity, MBFv, and PI were obtained as a mean of two best measurements chosen from each side; otherwise, the measurements from the available side only were used. In patients with atrial fibrillation, a cardiac cycle with the highest peak, which represents a more synchronized cardiac contraction and hence better cardiac output, was selected when measuring the velocities of arteries, as recommended (34, 35). As mentioned above, an average over at least 10 heart beats was applied in order to have a representative value of the TCD measures. All recordings were stored on a PC for the off-line analysis.

### Statistical analysis

Since the Shapiro normality test indicated that the most target variables were not normally distributed, a nonparametric analysis was carried out. Data were presented as as mean ± standard deviation for continuous variables, and as median, and first-third quartile, or number and percentage, when appropriate. As stated, the sample was divided into three groups according to WMLs severity. For inter-group analysis, the χ^2^ test and the Kruskal-Wallis test were used for comparison when appropriate, followed by Dunn post-test for multiple comparison. Correlation analysis between TCD parameters and severity of WMLs, depression (HDRS), and executive dysfunction (StroopT) on the whole sample was performed by means of the Spearman's correlation coefficient. A multiple regression analysis on TCD parameters (dependent variables) and the influence of demographic (age, gender, and education) and clinical variables (vascular risk factor, StroopT, and HDRS), and WMLs severity as predictors were computed. We applied a backward elimination stepwise procedure for the choice of the best predictive variables in according to the Akaike information criterion (AIC). Analyses were performed using an open source R3.0 software package. A 95% of confidence level was set with a 5% alpha error. Statistical significance was set at *p* < 0.05.

## Results

From a total sample of 84 participants, 8 (9.5%) were excluded because of bilateral insufficient/absent acoustic transtemporal window. Therefore, 76 consecutively patients (mean age 72.5 ± 5.3 years, 53.9% females) satisfying the inclusion criteria were recruited. Among them, an adequate acoustic window was found unilaterally in 3 subjects (4%).

Tables 1, 2 show clinical, demographic, neuropsychological, and TCD characteristics of the sample and the comparison between patients with different severity of WMLs. The neurological examination was unremarkable in all patients, except for diffuse brisk tendon reflexes in 8 patients and unsteadiness in 3. The mean depth of M1 tract of MCA providing the best TCD signal was 54 ± 4 mm. Moreover, at the time of the examination, mean heart rate was comparable between the three groups (69.0 ± 7.9 vs. 67.0 ± 8.2 vs. 65.0 ± 6.7 bpm, respectively), as well as the mean arterial pressure (97.0 ± 7.2 vs. 99.0 ± 6.8 vs. 99 ± 7.4 mmHg, respectively). Based on HDRS score, the depression was rated as: mild in 46 patients, moderate in 27 patients, and severe in 3 patients. According to WMLs severity, 20 patients were classified as Fazekas 1 (group I), 32 patients as Fazekas 2 (group II), and 24 patients as Fazekas 3 (group III). The three groups were comparable in terms of age (*p* = 0.43), education (*p* = 0.48), gender (χ^2^ = 1.63; *p* = 0.44), hypertension (χ^2^ = 0.54; *p* = 0.76), current smoke (χ^2^ = 1.09; *p* = 0.90), diabetes (χ^2^ = 2.13; *p* = 0.34), dyslipidemia (χ^2^ = 1.91; *p* = 0.38), coronaropathy (χ^2^ = 0.08; *p* = 0.96), atrial fibrillation (χ^2^ = 2.88; *p* = 0.24), and HDRS score (*p* = 0.14).

Table 1**(A)** Demographic, neuropsychological, and TCD features of VD patients and each groups according WMLs severity (Kruskal Wallis test); **(B)** significant differences and *post-hoc* analysis.

**Table:** 

**Variable**	**All patients** **(*n* = 76)**	**Group I** **(*n* = 20)**	**Group II** **(*n* = 32)**	**Group III** **(*n* = 24)**	***p-value***
**(A)**					
**Age** (mean ± SD)	72.53 ± 5.36	71.95 ± 4.55	71.81 ± 4.71	73.96 ± 6.71	0.43
**Education** (mean ± SD)	7.85 ± 3.89	8.20 ± 4.05	8.47 ± 4.36	6.75 ± 2.93	0.48
**HDRS** median (IQR)	16.00 (11.75–18.25)	13.50 (10.75–16.25)	15.50 (12.0–18.0)	16.50 (12.75–19.25)	0.14
**Stroop T** median (IQR)	51.90 (36.15–70.10)	49.85 (27.82–66.25)	47.45 (31.25–62.25)	59.70 (49.78–76.02)	**0.04**
**MBFv** median (IQR)	53.00 (51.00–55.25)	54.00 (51.75–56.25)	53.50 (52.00–56.00)	51.00 (50.0–53.0)	**0.007**
**PI** median (IQR)	0.96 (0.85–1.10)	0.81 (0.60-0.87)	0.96 (0.89-1.09)	1.10 (1.03-1.16)	<**0.001**

**Table:** 

**(B)**						
**Variable**	**Group I**	**Group II**	**Group III**	**Kruskal-Wallis test**	***Post-hoc*** **analysis**	
	**Median** **(I-III quartile)**	**Median** **(I-III quartile)**	**Median** **(I-III quartile)**	***p*** **value**	**Differences**	***p*****-value**
**Stroop T**	49.85 (27.82–66.25)	47.45 (31.25–62.25)	59.70 (49.78–76.02)	0.04^*^	I-II	0.70
					I-III	0.15
					II-III	0.04^*^
**MBFv**	54.00 (51.75–56.25)	53.50 (52.00–56.00)	51.00 (50.00–53.00)	0.007^*^	I-II	0.49
					I-III	0.01^*^
					II-III	0.02^*^
**PI**	0.81 (0.60–0.87)	0.96 (0.89-1.09)	1.10 (1.03–1.16)	<0.001^**^	I-II	<0.001^**^
					I-III	<0.001^**^
					II-III	0.01^*^

TCD, transcranial Doppler ultrasonography; VD, vascular depression; Group I, Fazekas score 1; Group II, Fazekas score 2; Group III, Fazekas score 3; WMLs, white matter lesions; HDRS, 17-item Hamilton Depression Rating Scale; Stroop T, Stroop Color–Word Test interference; MBFv, mean blood flow velocity; PI, pulsatility index; SD, standard deviation; IQR, interquartile range (I-III quartile); numbers in bold, statistically significant p values;

* p < 0.05;

** *p < 0.001*.

**Table 2:** Demographic variables and Vascular Risk Factor of VD patients and each groups according WMLs severity (χ^2^ test).

**Variable**	**All patients (*****n*** = **76)**		**Group I** ** (*****n*** = **20)**		**Group II** ** (*****n*** = **32)**		**Group III ** ** (*****n*** = **24)**		***p***
	***n***	**%**	***n***	**%**	***N***	**%**	***N***	**%**	
**GENDER**									
Male	35	46.05	7	35.00	15	46.88	13	54.17	0.44
Female	41	53.95	13	65.00	17	53.13	11	45.83	
**HYPERTENSION**									
Yes	63	82.89	16	80.00	26	81.25	21	87.50	0.76
No	13	17.11	4	20.00	6	18.75	3	12.50	
**DIABETES**									
Yes	18	23.68	3	15.00	7	21.88	8	33.33	0.34
No	58	76.32	17	85.00	25	78.13	16	66.67	
**DYSLIPIDEMIA**									
Yes	30	39.47	6	30.00	12	37.50	12	50.00	0.38
No	46	60.53	14	70.00	20	62.50	12	50.00	
**CORONAROPATHY**									
Yes	14	18.42	4	20.00	6	18.75	4	16.67	0.96
No	62	81.58	16	80.00	26	81.25	20	83.33	
**CURRENT SMOKE**									
Yes	25	32.89	5	25.00	10	31.25	8	33.33	0.90
No	51	67.11	13	65.00	22	68.75	16	66.67	
**ATRIAL FIBRILLATION**									
Yes	9	11.84	2	10.00	2	6.25	5	20.83	0.24
No	67	88.16	18	90.00	30	93.75	19	79.17	

*Legend: VD, vascular depression; WMLs, white matter lesions; Group I, Fazekas score 1; Group II, Fazekas score 2; Group III, Fazekas score 3; n, number of subjects*.

**Figure 1:**
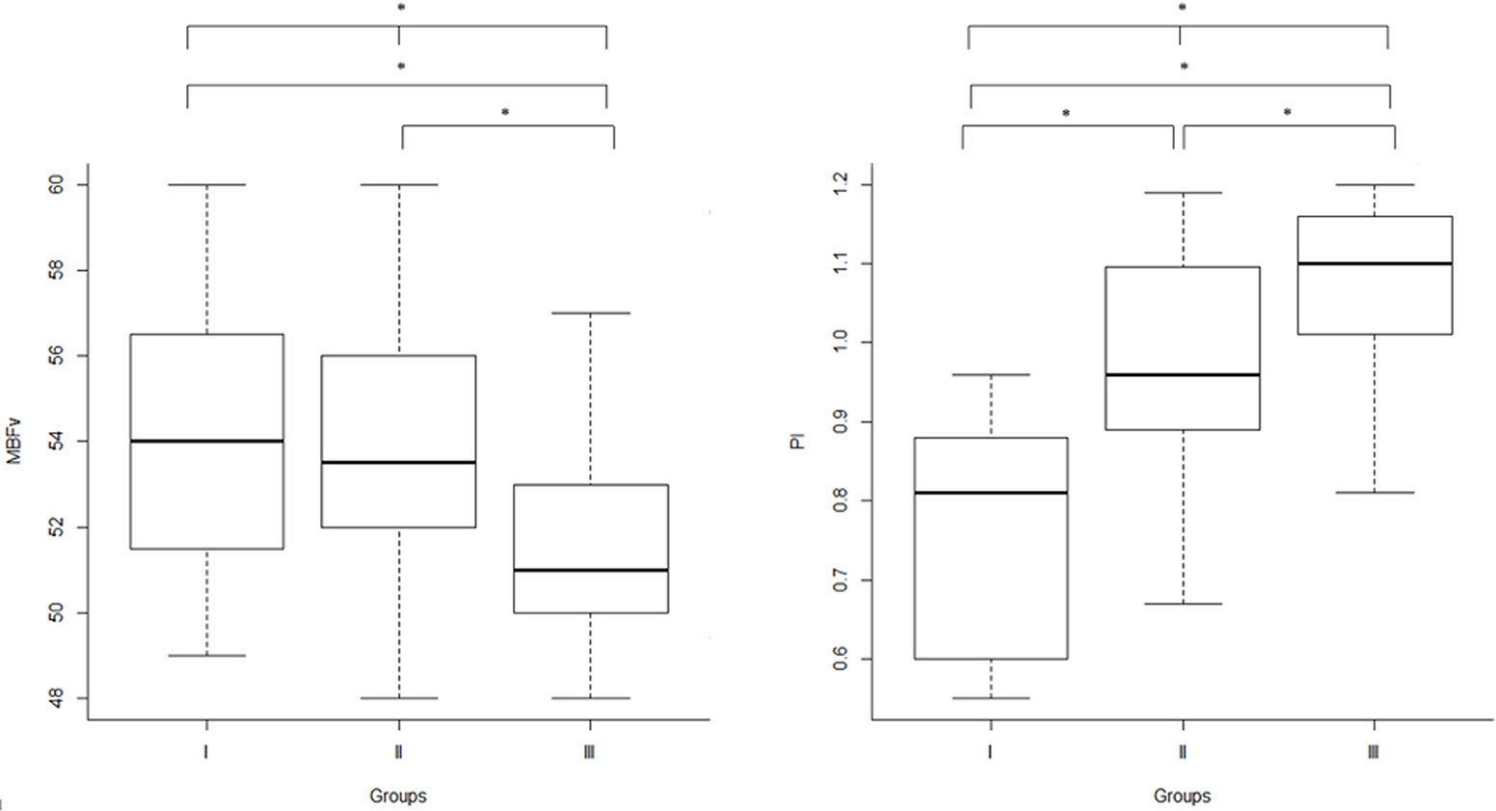
Inter-group analysis: TCD parameters (right: MBFv, left: PI) in the three groups, according to WMLs severity scored by Fazekas scale. TCD, transcranial Doppler ultrasonography; MBFv, mean blood flow Velocity; PI, pulsatility index; WMLs, white matter lesions; Group I, Fazekas score 1 (mild WMLs); Group II, Fazekas score 2 (moderate WMLs); Group III, Fazekas score 3 (severe WMLs); ^*^p < 0.05.

Regarding TCD measures and executive dysfunction, we found significant difference in MBFv (*p* = 0.007), PI (*p* < 0.001), and Stroop T (*p* = 0.04) between the three groups. In particular, PI was significantly higher in group III compared with the others (group III vs. group I: *p* < 0.001; group III vs. group II: *p* = 0.01), and in group II than group I (group II vs. group I: *p* < 0.001). MBFv was significantly lower in group III than group I (*p* = 0.01) and group II (*p* = 0.02). Group III exhibited a significant worse score of Stroop T than group II (*p* = 0.04) (Table 1, Figure 1).

**Figure 2:**
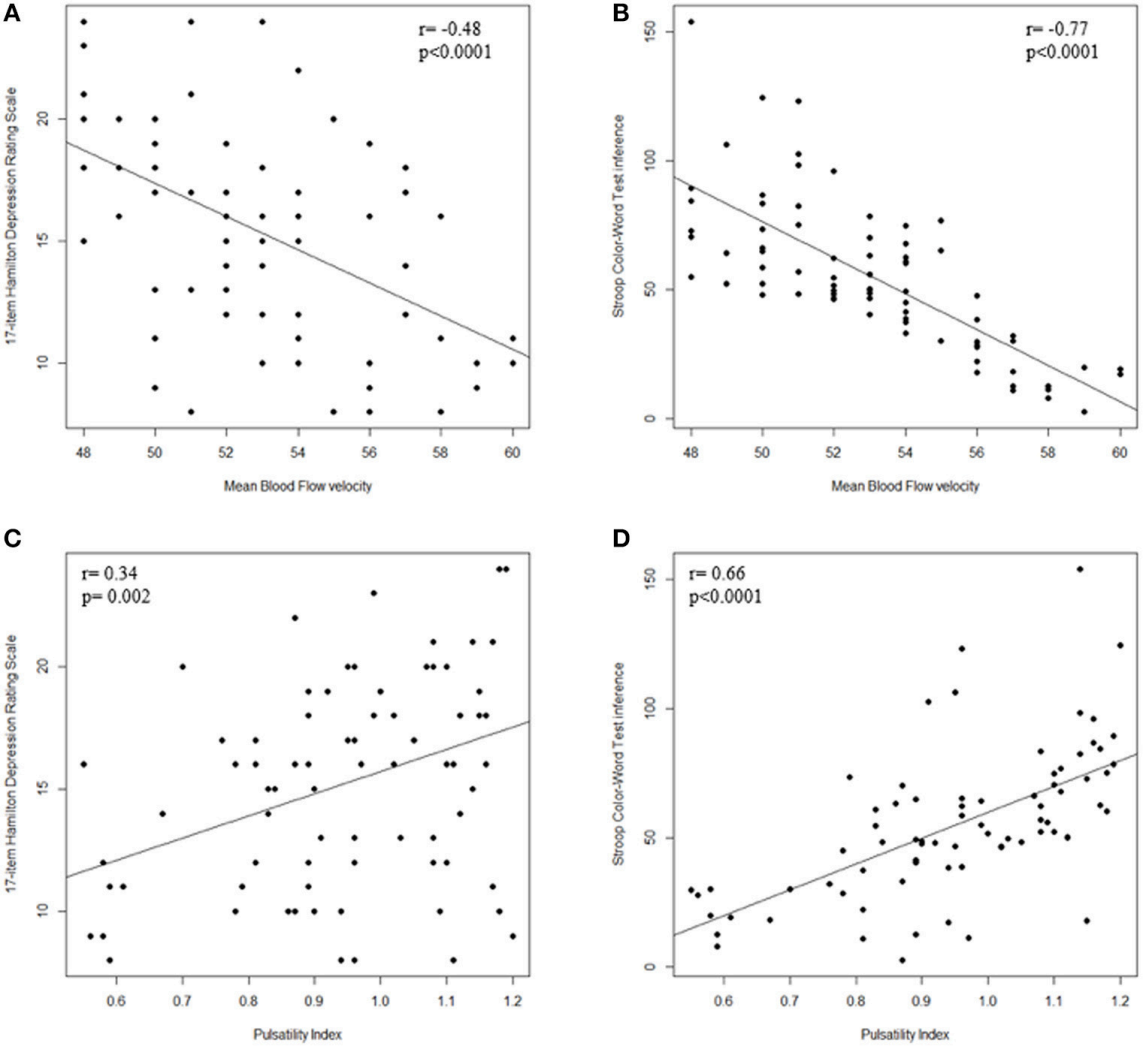
Correlation between TCD parameters and cognitive dysfunction. **(A)** Scatter plot of MBFv and HDRS; **(B)** Scatter plot of MBFv and Stroop T; **(C)** Scatter plot of PI and HDRS; **(D)** Scatter plot of PI and Stroop T. TCD, transcranial Doppler ultrasonography; MBFv, mean blood flow Velocity; PI, pulsatility index; HDRS, 17-item Hamilton Depression Rating Scale; Stroop T, Stroop Color–Word Test interference; WMLs, white matter lesions.

As shown in Figure 2, Spearman correlation demonstrated a significant: (i) negative correlation between MBFv and HDRS (*r* = −0.48; *p* < 0.0001) and Stroop T (*r* = −0.77; *p* < 0.0001); (ii) positive correlation between PI and HDRS (*r* = 0.34; *p* = 0.002) and Stroop T (*r* = 0.66; *p* < 0.0001); (iii) negative correlation between MBFv and WMLs severity (*r* = −0.34; *p* = 0.002); (iv) positive correlation between PI and WMLs severity (*r* = 0.64; *p* < 0.001).

As reported in Table 3, MBFv was significantly and independently associated with HDRS and Stroop T, whereas PI with both Stroop T and WMLs severity. A near significant *p* value (0.052) was found for WMLs severity as a predictor of MBFv. Given that psychomotor slowing seen in depression can affect cognitive performance, we have also used HDRS item psychomotor retardation as a correction factor, but no significant association was found with cognitive dysfunction.

**Table 3:** Backward linear regression: significant predictors of MBFv and PI.

**Dependent variables**	**Predictors**	**β**	**Std β**	***P***	**Adjusted R^2^**
MBFv	HDRS	−0.23	−0.32	<**0.001**	0.67
	Stroop T	−0.07	−0.67	<**0.001**	
PI	WMLs severity	0.12	0.54	<**0.001**	0.65
	Stroop T	0.003	0.47	<**0.001**	

*MBFv, mean blood flow velocity; PI, pulsatility index; HDRS, 17-item Hamilton Depression Rating Scale; Stroop T, Stroop Color–Word Test interference; WMLs, white matter lesions; numbers in bold, statistically significant p values*.

## Discussion

### Main findings

To the best of our knowledge, this is the first TCD study evaluating cerebral hemodynamics in patients with LLD and SIVD. The main result is that significant changes of the two main indexes of cerebral perfusion (increased PI and reduced MBFv) were found in patients with WMLs, executive dysfunction and depression. This finding supports the concept that LLD clinically presenting as depression-executive dysfunction is associated with vascular damage of the frontal-subcortical circuits implicated in mood-affect regulation and cognition (2, 36). In particular, the combination of a low perfusion state and a high level of vascular resistance suggests a diffuse cerebrovascular pathology which might arise from the small vessels and then extend to larger arteries.

As known, the subcortical white matter of the frontal-subcortical circuits is particularly vulnerable to hypoperfusion and ischemia: compared to the cortex, indeed, it is poorly vascularized by long penetrating and perforating arteries that give off short branches (37). Furthermore, the cerebral white matter is an arterial border zone, since the arteries perfusing it do not anastomose and are susceptible to infarction due to impaired autoregulation (38). As a result, hypoperfusion induces chronic damage within the white matter tracts, which, in turn, leads to an ischemic interruption of the frontal-subcortical circuits and to a secondary executive dysfunction and depression. In addition, it has been demonstrated that vascular dysregulation is common in LLD and CBF reduction can impair regional brain function, contributing to affective and cognitive symptoms (39). Thus, greater WMLs severity may be a marker of broader deficits in perfusion and autoregulation, as individuals with more severe WMLs exhibited reduced CBF in both white and gray matter regions (40). Accordingly, as showed by the correlation analysis, TCD parameters significantly correlated with WMLs severity and cognitive impairment, suggesting that these hemodynamic changes worsen as the vascular damage proceeds. Interestingly, when the three groups were compared, a significant difference in MBFv between group I and III (extreme grade of WMLs severity) and between group II and III (moderate vs. severe WMLs) was observed, although this was not between group I and II (mild vs. moderate WMLs); conversely, PI significantly differed between each group. In line with previous studies showing an association between TCD measures and WMLs load (41, 42), these findings suggest that reduced MBFv is more evident as the severity of SIVD advances, whereas change of PI may appear even in the early stage of the small vessel disease.

To date, the causal relationship between brain changes, progressive WMLs load, and LLD remains controversial, and current data propose that the term VD should be reserved for depressed older patients with depression of the late life and imaging evidence of cerebrovascular pathology (43). In this view, the multiple regression analysis showed an independent association between increased PI and both WMLs load and executive dysfunction, suggesting that PI might be considered as an index of cerebral microangiopathy also in patients with LLD and SIVD.

In addition, the present study showed that a reduced MBFv was significantly associated with both depressive symptoms and executive dysfunction. In this context, alteration of MBFv, which is extensively used as a proxy for CBF (44), occurs as a results of structural changes in the cerebral vessels and neurotransmission systems involved in the regulation of mood and cognitive function. Notably, the results were all adjusted for demographic variables and cardiovascular risk factors, indicating that microvascular dysfunction likely represents an independent factor in the development of LLD. Moreover, the finding that HDRS and Stroop T were significant predictors of MBFv, even after correcting also for WMLs severity, supports the independent role of cerebral hypoperfusion in LLD and suggests that altered MBFv might be considered as a marker of hemodynamics dysfunction and cognitive impairment in VD patients.

Recently, increasing evidences have focused on the role of hemodynamic changes in cognitive impairment and dementing disorders; in particular, different TCD profiles have been identified based on the type of dementia and the study design (16), also in the early stage of the disease (22), as well as in patients with cognitive impairment after transient ischemic attack and minor stroke (45). As known, elderly with geriatric depression are at higher risk of dementia (5, 46) and WMLs and executive dysfunction are both linked to poor response to treatment and the occurrence of chronic depression (47, 48). Thus, the early detection of a TCD pattern of hypoperfusion in a patient with LLD and SIVD should warn the clinician toward a careful identification and management of vascular risk factors and a strict monitoring for the risk of progression into an overt dementia. In this frame, since large arteries and microcirculation are communicating compartments, TCD flow spectra of the large cerebral arteries can disclose relevant information about the functional state of the downstream microcirculation.

### Limitations

The main limitation is the lack of a control group; however, the well-known difficulties in the recruitment of a comparable number of age-matched healthy subjects without any imaging evidence of SIVD (that is strikingly prevalent among elderly) and cognitive impairment at the neuropsychological evaluation should be taken into account. Second, although this sample may not be representative of the whole population of patients with LLD, it reliably represented VD patients given their clinical-psychocognitive and neuroradiological features (43). Furthermore, the sample was very homogeneous in terms of psycho-cognitive features and different severity of WMLs. Indeed, strict inclusion and exclusion criteria had to be used in order to screen a population of elderly patients with SIVD and depression-executive dysfunction, but without clinical and neuroimaging sign of a previous stroke, or ultrasound evidence of significant stenosis of intracranial vessels. Third, as usual in TCD research, patients with insufficient bone windows cannot be investigated, a finding that occurs bilaterally in approximately 10% of subjects. Nevertheless, although the findings from this study need caution before being generalized, the possibility that the subjects selection at baseline could have biased the results is unlikely, since this scenario would have been caused an underestimation of true associations. Fourth, TCD parameters were measured from the MCA only, which, however, perfuses the largest vascular territory of the brain, and is ideally located for TCD recording, providing satisfactory velocities. The anterior cerebral artery, although supplies part of the frontal lobe, has a Doppler signal not always sufficiently intense to allow reliable velocity determination (49). Finally, although VD is widely defined by previous research, it is still not a firm nosological category. Given that the causal relationship between vascular lesions and LLD remains controversial, further neuropathological studies are needed (43).

### Conclusions

This study highlights the role of TCD in the non-invasive, *in vivo*, and real time assessment of hemodynamics in LLD and SIVD. Given the cross-sectional nature of the present study, the early detection of cognitive deterioration requires a longitudinal follow-up of depressed patients. Therefore, further research is needed to confirm these findings, their modifications over time, and the clinical and TCD correlates of disease process and progress.

## Data availability statement

All datasets for this study are included in the manuscript.

## Author contributions

AB and RB conceived and designed the study; VP and LB organized the database, performed the statistical analysis, and reviewed the literature; GL dealt with psychometric evaluation; MC, LV, and MP wrote the manuscript; GP and RB critically reviewed and finalized the paper. All authors contributed to manuscript revision, read, and approved the submitted version.

### Conflict of interest statement

The authors declare that the research was conducted in the absence of any commercial or financial relationships that could be construed as a potential conflict of interest.

## Abbreviations

CBF: cerebral blood flow
DSM-5: Diagnostic and statistical manual of mental disorders-5th edition
HDRS: 17-item Hamilton Depression Rating Scale
LLD: late-life depression
MBFv: mean blood flow velocity
M1: proximal tract of the middle cerebral artery
MCA: middle cerebral artery
MMSE: Mini Mental State Examination
MRI: Magnetic Resonance Imaging
PI: pulsatility index
SCID-5: Structured Clinical Interview for DSM-5
SIVD: subcortical ischemic vascular disease
Stroop T: Stroop Color-Word Test interference
TCD: Transcranial Doppler ultrasonography
VaD: vascular dementia
VD: Vascular Depression
WMLs: white matter lesions.
